# Forage Consumption and Its Effects on the Performance of Growing Swine—Discussed in Relation to European Wild Boar (*Sus scrofa* L.) in Semi-Extensive Systems: A Review

**DOI:** 10.3390/ani9070457

**Published:** 2019-07-18

**Authors:** M. Jordana Rivero, Vicente Rodríguez-Estévez, Silvana Pietrosemoli, Cecilia Carballo, Andrew S. Cooke, Anne Grete Kongsted

**Affiliations:** 1 Departamento de Ciencias Agropecuarias y Acuícolas, Facultad de Recursos Naturales, Universidad Católica de Temuco, Temuco 4780000, Chile; 2 Rothamsted Research, North Wyke, Okehampton, Devon EX20 2SB, UK; 3 Department of Animal Production, University of Cordoba, 14071 Córdoba, Spain; 4 North Carolina State University, Animal Science Department, Raleigh, NC 27695, USA; 5 Departamento de Producción Animal, Universidad Politécnica de Madrid, Madrid 28040, Spain; 6 Departamento de Producción Animal y Pasturas, Facultad de Agronomía, Universidad de la República, Montevideo 11800, Uruguay; 7 Department of Agroecology, Faculty of Science and Technology, Aarhus University, 8830 Tjele, Denmark

**Keywords:** forage intake, grazing pigs, outdoor pig production, free-range pigs, grazing behaviour, sustainability

## Abstract

**Simple Summary:**

Outdoor-reared European wild boar (*Sus Scrofa* L.) is regarded as a delicacy by consumers due to its favourable meat properties and an association with high welfare standards. The rearing of wild boar on pasture has the potential to minimise input costs relative to conventional production systems. However, some pasture production systems have been found to perform poorly due to low growth rates. This review collates the available scientific evidence on pasture-based production of wild boar and domestic pigs, to identify factors that influence feed intake, performance, and behaviour. Factors explored include season/weather, dietary supplementation, grazing management, forage availability, herbage quality, and sward type. For example, the additional feed availability associated with pasture grazing has been shown to be a significant factor that positively correlates with dry matter intake of wild boar. This has been demonstrated to result in better feed conversion efficiency and reduced feed costs without reductions in growth rates compared to animals without access to pasture. Furthermore, the increased availability of favoured species in pasture may also promote dry matter intake. The long-term sustainability of wild boar production is dependent on the economic, social, and environmental viability of the systems. Pasture-based production systems may be one way by which this can be achieved, but only if implemented correctly.

**Abstract:**

Due to its distinct properties, wild boar meat is considered a highly desirable consumer product, in a market that is expanding. Outdoor production is also favoured by consumers who value animal welfare and environmental sustainability when choosing meat products. There is evidence that farms that include pasture for grazing typically have reduced feeding costs. Such production systems can also be more environmentally sustainable as the input (pasture) is inedible to humans, compared to conventional indoor systems, which use human-edible feeds (e.g., soya). However, some wild boar farms have performed poorly compared to those rearing other swine such as hybrid wild boar and domestic pigs. Diet is central to all livestock production and is likely a significant influencing factor of wild boar performance, both in terms of forage consumption and nutritional composition. Other factors may also influence performance, such as weather, behaviour and grazing management. Wild boar production systems hold their own intrinsic value in a growing marketplace. However, information gathered through the study of wild boar has external applications in informing outdoor domestic pig production systems to encourage the use of pasture as part of the habitat of domestic pigs.

## 1. Introduction

Wild boar meat has a unique taste and texture profile, making it a delicacy, particularly in Europe, but also across Asia, North America, and South America [1,2]. When compared to domestic pigs, wild boar meat has a higher degree of fatness and larger loin area, with darker, leaner, and less tender meat [1]. It also has a greater mineral content, especially of phosphorus, iron, iodine, and zinc [3,4]. The fatty acid composition of the intramuscular fat has a greater content of omega 3 and a preferential omega 3/omega 6 ratio [5].

In addition to meat characteristics, outdoor wild boar and pig production is appreciated by consumers who consider animal welfare an important matter when choosing products [6]. This selling-point gives outdoor production a commercial advantage over intensive systems. Production systems that include large areas of pasture for grazing can also lead to a reduction in the cost of feeding since pasture is considered the most economical feed for animal production [7].

An herbage-based diet suits the omnivorous nature of wild boar [8,9] and has the potential to improve the nutritional quality of meat through increased levels of polyunsaturated fatty acids [10]. Moreover, the proportion and composition of the ingested forage affects the antioxidant and fatty acids composition of muscles, which, in turn, affects the contents of α-tocopherol, total phenols, hydrophilic and lipophilic antioxidant activity, and fatty acid profile [11]. Outdoor pig production systems based on high nutrient intake from pasture are considered more sustainable compared to indoor systems that are typically dependent on high inputs of cereals and soya [12,13]. Such crops are fit for human consumption and the feeding of them to animals represents an inefficient use of the resources used to produce them. Furthermore, there is an additional environmental impact of the processing, packaging, and transportation of these feeds. In contrast, some pasture production systems can contribute to maintaining certain agroforestry ecosystems; for example, the dehesa agrosystem (clear forest of evergreen oaks, *Quercus rotundifolia*) [14]. Such integrated approaches allow farmers to take advantage of the natural ecosystem, whilst retaining its intrinsic value.

Semi-extensive systems are an example of such pasture-inclusive production, where diet is based on areas of pasture for grazing and a supplementary diet is provided to satisfy the requirements of growing animals [15]. However, commercial farms producing wild boar often present poor performance indicators compared with those rearing hybrid wild boars or other pig breeds [16]. The target daily live weight gain for wild boar is typically 150–200 g/d, however, semi-extensive farms regularly fall short of this [17] and age at target slaughter weight (45–50 kg) is typically six to seven months. This low growth rate can be mainly attributed to genetic growth potential since the wild genotype of swine has evolved to survive in limiting environments, therefore faster growth rates and greater nutrients requirements might represent a disadvantage [6]. However, as an omnivorous and opportunistic species [8,18,19], wild boar can forage low quality and low-density feed resources whilst awaiting better resources (e.g., acorns and nuts) and then exhibiting a late-life compensatory growth when feed is more abundant [20]. The potential variety of wild boar diet can be advantageous to farmers, providing flexibility in the feed and land provided to the animals and allowing for the utilisation of resources (feed and land), which would otherwise hold little or no commercial value.

Diet quantity and quality are driving factors affecting wild boar performance under commercial systems. The objective of this review was to collate the scientific information available for grazing wild boars complemented with that for grazing domestic pigs. This combined approach is due to the intrinsic similarities between phenotypes but also due to the scarcity of information from the wild phenotype, to determine the factors influencing pasture consumption and its effect on the performance of *Sus scrofa* L. Intake of nutrients from pasture by domestic pigs has been extensively reviewed in 2003 [10] with the main emphasis on intakes by sows and growing-finishing pig offered supplementary concentrate ad libitum. The current review includes recent studies of growing-finishing pigs and wild boar offered a limited level of supplementary concentrate to complement the herbage consumed by grazing pastures.

## 2. Description of the European Wild Boar

The European wild boar (*S. scrofa* L.) belongs to the same species as domestic pigs (*S. scrofa domesticus*). Although it can be reared in captivity, the wild boar phenotype is suited to the wild habitat, since artificial selection has not operated to the extent that it has with domestic pigs. Natural genetic selection within wild boar populations has been driven by selection pressures of challenging environments, factors such as food availability, and predation. The resulting phenotypes are not necessarily growth/production orientated, as they are in commercial systems where those selection pressures are not present.

Wild boars, which are monogastric ungulates [18], are characterised by having a fore section and head more developed than domestic pigs, erect ears, and fangs in the lower jaw [21]. The average birth weight is approximately 0.9 kg per piglet [22]. They present very pronounced sexual dimorphism with clear size differences between sexes when mature (above 24 months of age). Males are typically 5%–10% larger and 20%–30% heavier than females (males: 50 to 130 kg, females: 45 to 90 kg [23]), although no differences are observed in the youths (7–12 months) or in the sub adult stage (13–24 months) [24]. Moreover, adult size and weight are largely determined by environmental factors.

Wild boar have certain internal organs, such as the liver and heart, that are larger than in the domestic pig [25], whilst organs such as the small intestine are smaller (length, area, volume, and weight) [26]. Wild boar also typically have a thinner layer of back fat than domestic pigs [27]. Comparing the gastrointestinal morphology of Vietnamese wild boar and a local heritage breed (Muong pigs), Trang et al. [28] reported that the wild boar had a larger stomach, with a more globose and rounded shape, with greater capacity for voluminous feed storage, formed with a lesser proportion of cardiac glands, a greater proportion of proper gastric glands and with no differences in the proportion of pyloric glands.

Wild boar can utilise nutrients from fibrous food through microbial fermentation in the caecum and colon [29,30]. Although the differences are minor, wild boar would have a greater capacity to digest organic matter (OM) and neutral detergent fibre (NDF) than the domestic Meishan pig [31]. According to Elston and Hewitt [32], this capability can be attributed to a larger body mass and colon, a longer food retention time, and therefore more microbial fermentation in the colon. These authors found that the rate of the fibre passage was similar to the rate registered for foregut fermenting species. This efficiency in fibre digestion is also observed in Uruguayan heritage breed pigs (Pampa Rocha breed); these pigs have a more developed digestive system when they are grazing [33], showing adaptation to the consumption of fibrous foods [34]. However, Hodgkinson et al. [35] observed that wild boar obtain 20% less digestible energy (DE) from fibre-rich foods (alfalfa meal with 50.4% NDF) than domestic pig (Landrace × Large White).

As an opportunistic and omnivorous species, wild boar diet is determined to a large extent by the spatial and temporal availability of different foods. Nevertheless, they have a preference for vegetables [36], which comprise 86%–96% of the dietary volume consumed by wild boar in their natural environments [8,9,37,38,39]. Skewes et al. [40] reported that grass leaves were found in 75% of the wild boar stomach content samples analysed. Moreover, Groot Bruinderink et al. [41] found that the diet of wild boar contained a large proportion of grasses (40%–60%), especially during winters when mast (the generic name for nuts, acorns, seeds, buds, or fruits of trees and shrubs that are eaten by animals) availability was low, and that during spring most of the vegetable fraction is obtained by grazing [42]. A study by Schley and Roper [8] showed that agricultural crops represent an important component of wild boar diet. The geographical and seasonal variations reflect the species adaptation to local and seasonal food availability [42].

## 3. Characterisation of Production Systems

The rising consumer demand for healthier meats, produced in more sustainable systems [43,44] has opened the door to wild boar producers who are also able to take advantage of the emerging exotic-meat/game-meat niche market, and the nutritional and sensory characteristics of wild boar meat [1,45,46]. Cooper and Van der Merwe [47] describe the potential benefits of game production due to a high suitability of animals to their environment. This reduces operational capital requirements because the environment does not need to be changed or managed as significantly as it may for domestic breeds. Furthermore, the population may already have developed resistance or resilience against pathogens within that environment, reducing associated welfare and economic costs.

This also opens up a potential revenue source through agri-tourism activities. Worldwide, wild boar are mostly managed using free-range practices, which takes advantage of wild boars’ capacity to utilise the different feedstuffs available [48]. Similar to wild boar, most heritage pig breeds also show adaptation to the local environment and less dependence on external resources than selected breeds [49,50,51,52].

Broadly speaking, there are three commercial systems to produce wild boar:

- Semi-extensive—Animals have access to pasture for grazing and they are given a supplementary diet [17,53,54], which is similar to outdoor pig production systems [44].
- Semi-intensive—The area per animal is smaller than that of the semi-extensive systems, large outdoor pens are used, and grains, by-products and hay are supplied [15,21].
- Hunting game estates—Most commonly found in Europe, their populations have increased significantly in the last quarter of the 20th century [23,55], a change that has been largely associated with the widespread practice of supplementary feeding, reforestation, intensification of crop production, and mild winters. In Europe, hunting is the main origin of wild boar meat while farming systems that include grasslands for grazing are predominant in countries like the UK or Finland [56].

### Growth Rate as a Limiting Factors in Semi-Extensive Wild Boar Production Systems

The main limiting factor to wild boar production is low growth rates. The superior performance of domestic pigs has been attributed to a greater postnatal protein accretion and myofiber hypertrophy, a consequence of historic selective breeding for lean growth [57]. The target growth rate for the wild phenotype is 150 to 200 g/d, however, observed values range from 94 to 402 g/d [16,21,22,24,31,58] compared to an average of 800 g/d in domestic pigs [59]. Lösel et al. [25] observed that, at similar ages (94 ± 3 d), the live weights (LW) of domestic pigs, fed a commercial diet ad libitum, were greater (47.3 kg) than those recorded for wild boar (12.7 kg) on the same treatment, a trend also observed by Wang et al. [16]. Looking exclusively at wild boar, Watthanakum [60] found that, under intensive management, growth rates were significantly higher for male piglets (270 g/d) compared to female piglets (203 g/d).

Despite poor real-world performance, average daily gains (ADG) of between 227 and 303 g/d have been achieved under experimental conditions [53,61,62,63]. Recently, Hodgkinson et al. [6] compared European wild boars and domestic pigs in a 60-d study under semi-extensive management and found that wild boar grew at a rate of 233 g/d, while the ADG of pigs was 758 g/d. Wang et al. [16] compared the growth performance of wild boar over 100 d with Bamei (heritage Chinese breed), Yorkshire (improved modern breed), and their crosses fed ad libitum and found that domestic pig breeds and the hybrids showed greater ADG than wild boar. Moreover, Watthanakun [60] concluded that wild boars reared as domestic pigs have a conversion rate of 3.9, a very poor feed efficiency compared to the NRC [59] pig recommendation (feed: gain <3).

There are two root causes for slow growth rates, genetics, and the environment. Quijada and Hodgkinson [64] investigated the genetic component and tested four diets containing different levels of digestible energy (2.2, 2.4, 2.6, and 2.8 Mcal DE/kg), with appropriate levels of lysine. They observed that while some animals showed lean tissue growth of around 70 g/d, others did so at a lower rate of 20 or 30 g/d. Furthermore, animals with similar initial weights and receiving the same diet (within the same treatment), presented high levels of variability in gains and losses of lean tissue, with coefficients of variation greater than 100% for lean tissue deposition and up to 92% for ADG. A large individual variation in pasture consumption has also been observed [53], which could indicate that there is great genetic variability in *S. scrofa* L. that could be exploited through breeding programs [65]. To improve growth rate and carcass performance some farmers have begun cross-breeding wild boar with domestic pigs within their breeding programme, known as hybrids [66].

Abiotic environmental factors, such as temperature or radiation, are impractical to control in an outdoor production system. However, diet is a controllable variable on-farm, and in semi-extensive systems, it is typically composed of dietary supplements and grazed forage. For wild boars reared in semi-extensive systems, Quijada [67] determined lysine concentration, optimal DE, and optimal lysine/DE ratio in the supplementary diet to maximise the accumulation of lean tissue and without over-fattening. This was determined to be 8 g lysine per kg dry matter (DM), 3.34 Mcal DE/kg DM, and 2.39 g lysine/Mcal DE. Growing wild boar can obtain an average of 52% and 142% of their DE requirements for maintenance through forage consumption, in summer and spring, respectively [68].

Crossbreeding wild boar with modern pigs has the potential to improve performance and production, whilst retaining some of the desirable traits of wild boar meat [69,70,71]. An economic analysis conducted in Argentina and Canada showed that the production, processing, and marketing of wild boar and hybrids of wild boar × domestic pig is a commercially viable venture [46,72,73].

Moreover, although Uruguayan heritage breed pigs have poor productive and reproductive indicators compared with more commercial pigs [74], their adaptation to the consumption of grazed forage and other foodstuffs available in the area represents a viable alternative to reduce feeding costs, therefore improving profit margins [75,76]. Furthermore, it has been shown that feeding with grazed forage can improve feed conversion ratios [76], that outdoor production systems (with pasture) generally require less investment in facilities [50], and that there is less dependence on external resources than indoor systems, which promotes system resilience [77].

## 4. Foraging Behaviour as Affected by Season and Resources Availability

Foraging behaviour of wild boar and domesticated pigs with access to pasture has been evaluated in a range of studies presented in Table 1 and discussed in the following.

Grazing is an important natural behaviour for wild boar, feral pigs (a domestic pig that has escaped into the wild and lives as a wild animal), and domestic pigs. Iberian pigs maintained in semi-natural conditions spend more than 50% of their time in foraging activities such as grazing [51] and are completely dependent on foraging natural resources. Rodríguez-Estévez et al. [78] observed that fattening Iberian pigs spent 61.5% of their time grazing (369 ± 7.8 min from 8:30 to 18:30). Likewise, in a natural environment, wild boar spend most of their time feeding (59.8%) or moving (27.4%) [79]. Ameneiros [80] reported a seasonal change in post-weaning behaviour of outdoor reared piglets (Pampa Rocha heritage breed); they spent more time grazing and exploring during spring (64%) than in winter (53%), and nose ringing did not affect animal performance.

Mast represents a sustainable resource for feeding finishers pigs in extensive systems [81]. Mast can be divided or classified into two main types, soft (berries) and hard (nuts and acorns). For wild boar and pigs, the hard mast is the most important type, especially in winter, due to its high energy density [23,51,82]. Seasonal variation in food availability (both type and amount) significantly influences foraging behaviour. During spring most grazing is above ground, but this shifts towards subterranean foraging (e.g., of bulbs and roots) during winter [48]. When foraging acorns in the dehesa with low stocking rate (≤1 pig/ha), Iberian pigs (not snout-ringed) rooted 69.6% of the days, with 4.4 ± 3.3 episodes of rooting per day. Rooting episodes disturbed a total soil daily area of 0.6 ± 0.8 m^2^ distributed in as many points as episodes [83]. The availability of water in the grazing plots is a factor that can affect both the time of grazing and the intake itself; a lack of water can increase the fresh grass intake and reduce DM intake from other resources [51].

Wild boar are predominantly active during twilight and at night, although diurnal activity is also observed when human disturbance is low. In captivity, activity is concentrated around the time of feeding events and a rest period typically occurs at noon, depending on weather conditions [99]. Significant correlations between the average daily air temperature and the time dedicated to grazing (r = −0.68) and to “wallowing” (inside water troughs) activities (r = 0.71) were found in summer by Rivero et al. [62]. In a semi-extensive system set-up, snout-ringed wild boar with access to pasture for 8 h a day spent on average 26% of their time grazing and 17% rooting, i.e., they dedicated a total of 43% of the time on pasture to foraging activities (grazing plus rooting) in autumn [100]. In summer, the time spent in grazing activities reached 42%, particularly concentrated in the first 3 h on pasture, with no rooting activity occurred, most likely due to a drier and harder soil [62]. This is supported by recent research by Hodgkinson et al. [6] who found that during summer snout-ringed wild boar spent over 40% of their time grazing, whereas domestic pigs spent only around 13% of their time grazing under the same system. From a practical point of view, shortening the grazing period to the first three hours of the morning could be an effective alternative to mitigate wild boar damage to pasture by trampling or rooting, taking advantage of the daily grazing pattern observed in wild boars. As rooting implies energy consumption, another option to reduce it could be to offer ad libitum feed to promote optimal foraging behaviour.

The wild boar rooting behaviour and its ability to prey on ground-nesting birds are the main reasons it is considered a pest [19]. Lombardini et al. [101] found that in Sardinia (Italy) wild boar damage events to agricultural crops is most prevalent in summer and early autumn, with the lowest incidence occurring in spring. This is the result of a seasonal adaptation of the wild boar diet in response to the available feed resources. Hence, in areas with winter mast, damage would be least prevalent during winter.

Rivero et al. [62] found a significant difference in the time dedicated to “playing” (usually including running after the partner or interacting in physical contact with the partner), where animals in the continuous stocking system (bigger grazing area) dedicated more time on friendly interactions than in a rotational grazing system (smaller grazing area), which might have animal welfare implications. Nevertheless, the grazing system did not affect the time dedicated to grazing by these animals. On the other hand, the plant species present in the sward were found to influence grazing time; Rachuonyo et al. [102] observed that pregnant sows dedicated more time to grazing and that the percentage of bare soil left after grazing was greater on the legume swards compared with grasses. Similarly, Jakobsen et al. [87] observed that free-range pigs (not snout-ringed) spent more time grazing a lucerne (*Medicago sativa* L.) sward compared with a grass sward.

## 5. Factors Affecting Forage Dry Matter Intake

Wild boars’ daily feed intake typically varies between 3% and 5% (dry matter basis) of their live weight [103]. Forage intake in wild boar and in domesticated pigs with access to pasture has been quantified in a range of studies presented in Table 1 and discussed in the following.

Individual differences in forage consumption by snout-ringed wild boar has been observed [53], varying on average from 210 to 550 g DM/d per animal (18.8 ± 0.8 kg LW on average) depending on the season and the dominant plant species (variation coefficients ranged from 108% to 137%). Forage consumption from pasture represents between 26% and 27% of the total DM consumed [62,63], and wild boar obtain a significant proportion of DE from grazed pasture (52%–53% of DE for maintenance [63,68]).

In relation to pasture consumption of domestic pigs, Mowat et al. [95] reported an average of 100 g/d OM from a *Lolium perenne* and *Trifolium repens* mix sward, for pigs in spring (50–60 kg LW) when supplementary diet was offered ad libitum. The authors indicated a low variability among individual animals and a forage consumption of only 4% of the total OM consumed, contrasting with the 15%–20% DM reported for not snout-ringed growing pigs foraging lucerne (average LW 58 kg) and with restricted access to supplementary diet registered by Jakobsen et al. [87]. Bauzá et al. [85] found that pigs (30–60 kg LW) with a restricted supplementary diet consumed an average of 372 g/d DM of pasture, results complemented by those of Jakobsen et al. [87], who reported feed intakes of 330–470 g/d DM for growing pigs. Rodríguez-Estévez et al. [51] found comparable DM intakes of 380–490 g/d in snout-ringed Iberian finishing pigs, which were fed on a natural acorn-based diet [96]. Gustafson and Stern [86] reported a forage consumption (*L. perenne* and *T. repens*) of circa 200 g DM by pigs (30 kg LW) whilst Carballo et al. [104] observed daily forage consumptions between 97 and 208 g DM from snout-ringed Pampa Rocha pigs with average LW between 25 and 40 kg, respectively (with a concentrate restriction of 15%). In a similar experiment, Castro [105] observed daily pasture consumption of 424 g DM from creole pigs (average LW = 27.8 kg) managed with the same restriction level, highlighting the remarkable variability in forage consumption observed in swine. Recently, Hodgkinson et al. [6] found that, on a metabolic bodyweight basis, the forage DM consumption by wild boar over doubled that of the domestic pigs (25% vs. 10% of their total DM intake).

As mentioned previously, wild boar behave as optimal foragers; hence, depending on the foraging area, they always consume at least one energy-rich plant food such as mast (acorns, beechnuts, chestnuts, pine seeds, and olives), cereal grains, or other crops [8]. A direct in situ study, observing ingestive bites taken by continuously monitored Iberian pigs while foraging mast in the dehesa, showed a daily intake of 56.4 ± 2.3 MJ ME from acorns and grass, of which acorns accounted for 90.4% [96], while Sáenz de Buruaga [79] found that acorns were 87.1% of the wild boar DM intake in autumn–winter in northern Spain. In agricultural areas, wild boars mostly graze maize and other crops, like wheat, barley, and alfalfa [8,106].

### 5.1. Effect of Supplementary Diet Level

In addition to LW and age, Edwards [10] states that pasture intake by domestic pigs depends to a large extent on the level of supplementary diet supplied. Danielsen et al. [107] confirmed that observation and found that domestic pigs fed a restricted (30% reduction) supplementary diet consumed 19% more forage than pigs with free access to supplements. However, this increase in forage intake was not enough to compensate for the deficit of supplementary diet, and the ADG decreased by 11% when the supplementary diet was restricted. These results agree with that reported by Battegazzore [76], who recorded daily average forage consumptions of 0.90 and 1.53 kg DM/d for fattening pigs subjected to restrictions of 70% and 50% on concentrate allocation, respectively, where animals with greater restriction had a lower LW gain (0.53 vs. 0.65 kg/d) and better supplementary feed conversion efficiency (2.64 vs. 3.14 kg feed/kg LW). Rodríguez-Estévez et al. [51] observed a daily DM intake of 9.5%–13.7% from natural grass as a percentage of total DM intake for free fattening snout-ringed Iberian pigs foraging acorns without any restriction in the dehesa. According to Bauzá [108], growing pigs (30–60 kg LW) grazing *Lolium multiflorum* pastures with restricted supplemental feed (up to 20% reduction) showed pastures intake of 370 to 385 g DM/d, which represent 17% to 21% of the total intake, while the intake for finishing pigs (60–100 kg LW) was estimated to be 700 to 800 g DM, equivalent to 25% to 30% of the total intake.

Wild boar have been shown to obtain a greater proportion of its digestible energy for maintenance from forages than domestic pig [10,53]. Whilst wild boar cannot live entirely on a grass diet, and therefore require supplementation, it is possible that the LW benefits of dietary supplementation could be greater for pigs than for wild boar. Conversely, the relatively high growth rate of domestic pigs may be at, or near, its peak rate. Therefore, there may be more room for gains in growth rates in wild boar populations and this may be realised through positive intervention methods such as improved supplementation. Rivero et al. [63] found no differences in concentrated diet consumption among snout-ringed animals with no access to pasture (fed concentrate once a day) and grazing animals. Groot Bruinderink et al. [109] did not find significant differences between the amount of plant material in the stomach contents of wild boar that did or did not receive supplementary feeding. Similarly, Kanga et al. [88] concluded that forage consumption depends on herbage characteristics and not on the nutritional value of the supplementary diet.

### 5.2. Effect of Season (Including Environmental Temperature)

In natural conditions, season exerts a marked effect on wild boar feed intake. Their diet varies seasonally, with greater consumption of grasses and leaves in spring, roots in summer, mast and roots in autumn, and mast, roots, and leaves in winter [109,110]. Season not only impacts on feed availability but due to its influence in the phenology of the plants and their nutritive value, seasonality also affects consumption patterns [48]. An average pasture consumption of 484 g DM/d has been reported for spring [53] and between 106 and 242 g DM/d for summer [53,62,63]. According to Treyer et al. [111], wild boar DM intake is affected by photoperiod, with greater intake and fat tissue accretion occurring during the shorter days after the summer solstice.

Lebret [112] suggested that an ambient temperature over the higher critical temperature leads to decreased feed intake and consequently decreased growth rate in domestic pigs. In contrast, ambient temperatures below the lower critical temperature could lead to divergence of energy resources towards temperature homeostasis inducing greater feed intake accompanied by declining growth rates. Negative correlations were observed between average maximum daily air temperature and DM consumption: pasture intake r = −0.40, supplemental diet intake r = −0.52, and total DM intake r = −0.49 [63]. Similarly, despite domestic pigs not showing a marked reproductive seasonality as observed in wild boar, they are affected by high temperatures, which is a fundamental aspect to be considered in outdoor systems [113].

### 5.3. Effect of Pasture Type and Quality

The nutritional composition of feed is a significant factor influencing performance and health of swine. Herbage-based diets that are rich in fibre are consequently of low nutritional value because swine can only partly degrade dietary fibre. As a result, these diets have been associated with reduced nutrient utilisation and animal performance. The extent of these negative effects is influenced by the fibre source, type, and inclusion level [114]. However, herbage consumption may contribute significantly to overcome protein shortages caused by some diets (e.g., acorn-based diets of Iberian pigs in the dehesa) and grass may improve the profile of the amino acid supply to pigs [115]. As a direct effect of this amino acid supply, the retention of N is increased. However, this increase is below the maximum potential of protein gain for Iberian pig. To our knowledge, there is no available information on the value of fresh herbage as a source of digestible amino acids for the wild boar.

García-Valverde et al. [116] found that the intake of freshly cut herbage by Iberian pigs, as a complementary feed to acorns, resulted in a significant transfer of digesta from the small intestine to the hindgut, a significant reduction in the apparent ileal digestibility of nutrients and energy, and a moderate decrease in digestibility along the whole tract. However, van Wieren [31] did not find any significant relationship between dietary NDF content and the voluntary consumption of growing wild boars. According to the author, this could happen because at high NDF concentrations diets become non-palatable for swine, resulting in decreased consumption. Alternatively, NDF concentrations may be so high that it is not possible to increase consumption due to size limitations of the digestive tract. This lack of relationship between NDF content and forage voluntary intake of growing wild boars was also found by Hodgkinson et al. [53]. On the other hand, Allende et al. [117] reported that increased amounts of alfalfa forage in wild boar diets decreased the voluntary forage intake due to the increased NDF content of the diet.

### 5.4. Effect of Pasture Allowance and Herbage Mass

In pigs, pasture can contribute to the nutritional needs in a variable way, and that that contribution will depend on the type of forage and its allowance [10]. Andresen and Redbo [118], studying the grazing behaviour of growing pigs, concluded that the observed increase in consumption frequency could be related to the quantity and quality of forage offered. In growing wild boars, Hodgkinson et al. [53] found a positive correlation (r = 0.59) between herbage DM mass and apparent DM forage consumption. Moreover, Rivero et al. [63] studied the effect of herbage allowance on forage DM consumption by snout-ringed wild boars and found that the greater the forage DM available per animal the greater the DM consumption (more than double from low to high herbage allowance) with no detrimental effect on concentrated diet consumption or ADG.

### 5.5. Selective Consumption

Animals can demonstrate a selective consumption of certain components of the sward, such as particular species or plants with some specific characteristics. For instance, Groot Bruinderink et al. [109] found that wild boar prefer broadleaved plant species. The diet that animals select to consume in restrictive grazing conditions (variation in abundance or height of the species, field slope, etc.) generally differs from what they would consume if they were given complete freedom of choice [119].

Ballari and Barrios-Garcia [9] emphasised the role of wild boar energy requirement on food resources selection. Wild boar will select easy to digest forages with a low content of structural carbohydrates and greater protein content [48]. The attributes that characterise most desirable plants or their parts include high content of simple sugars, starch, and lipids and a low content of defence metabolites [48]. According to Stuth [120], animals seem to select few plant species and focus their choice on species that offer the maximum amount of green forage per bite. According to Rodríguez-Estévez et al. [78], that is the reason why Iberian pigs, an ancient breed, prefer to sight out or reject different acorns during the montanera (acorn) season, suggesting that pigs learn to use visual stimuli when grazing. This selective consumption is complementary to the theory of Optimal Foraging [78]; a foraging strategy that provides the most benefit (energy) for the lowest cost, maximising the net energy gained.

Information regarding the extent to which pigs select plants of different species, or different parts within a plant, when offered a mixed sward, is scarce [10]. Mowat et al. [95] studied individual consumption of grazing pigs and found that only one pig out of six showed selectivity for some species, i.e., towards *T. repens* in a mixed grass/legume sward. Sehested et al. [121] observed changes in the botanical composition of a *L. perenne* and *T. repens* mix sward, which could suggest a preferential grazing of *T. repens*. For snout-ringed finishing pigs raised outdoors, Battegazzore [76] observed a higher percentage of use of *Chicorium intybus* (79%) compared with *Trifolium pratense* and a grass species. These differences were higher when concentrate restriction levels were increased. The selectivity towards *C. intybus* was confirmed by Carballo [84] who also observed that pigs of lower LW are more selective and exerts a lower utilisation of the pasture than heavier animals. Moreover, Carlson et al. [122] observed a great ability of pigs to discriminate between leaves and stems of forage plants. The authors recorded a selective consumption of the high-sugar components of pasture cuttings of a *T. repens* and grass mix sward by growing pigs. Stern [86] postulated that grazing pigs are able to select plants or parts of plants, so the quality of the forage consumed differs from the general quality of the pasture; forage consumed was observed to be 8.5% higher in gross energy, and 26.7% and 23.8% lower in NDF and ADF, respectively, than forage on offer [63].

Working with snout-ringed wild boar, Hodgkinson et al. [53] found no differences in DM or nutrients intakes (growing animals, 18.8 kg initial LW) between two contrasting swards of *L. perenne* and *Plantago lanceolata*. However, when the two plant species made up a mixed sward, wild boar selected *P. lanceolata* [123]. The authors concluded that leaf length would influence the selective consumption of *P. lanceolata* over *L. perenne*, since there was a strong positive relationship between leaf length and grazing probability, and animals preferred longer leaves. Nevertheless, wild boar are able to subsist on shorter swards than larger animals [124].

Rachuonyo et al. [102] worked with pregnant sows and concluded that they preferred to graze *T. repens* and *M. sativa* (both legumes), and rooting on *T. repens*, in comparison with *Festuca arundinacea* and *Buchloe dactyloides* (both grasses). These authors attributed this preference to the greater palatability of the legumes, expressed in their succulent nature and ease of grazing, as opposed to grasses, which are more fibrous and harder to harvest due to their higher breaking force [121]. Grazing preference of European wild boar has been studied for the first time by Rivero et al. [125]. These authors found that wild boar prefer to consume herbage from taller plants (≈18 cm) than from shorter plants (≈12 cm) and display a partial preference for legumes over grasses; the grazing probability of legumes was 0.67 vs. 0.46 for grasses, and the defoliation frequency was also greater in legumes than in grasses (1.55 vs. 1.17). Therefore, a high-quality legume species would be a favourable companion species within the pasture [125] when an improvement on forage consumption is pursued. This could lead to increased forage consumption and consequently greater daily weight gains.

## 6. Effects on Animal Performance

Consumption of nutrients from pasture by grazing pigs depends upon the voluntary intake and the pasture nutritional value [10]. For growing domestic pigs the maximum value of forage inclusion is recommended to be 20% of total DM [122] since greater values will limit energy consumption and consequently growth [108]. However, Rivero et al. [63] concluded that forage inclusion above 20% in the diet of wild boar has no detrimental effects on the ADG or the feed conversion efficiency, and Rodríguez-Estévez et al. [96] registered 790 g/d of mean ADG for Iberian pigs fed only acorns and grass.

A decrease in supplementary diet consumption is to be expected with increased forage consumption due to the digestive tract distension, which would reach a limit, representing a process of substitution of supplementary diet for forage. Leite et al. [92] observed an average decrease of 15% in the supplementary diet consumption, a decrease in back-fat thickness of 21% and a decrease of 16% in ADG on snout-ringed pigs grazing *T. repens* compared with confined pigs (without access to pasture). In the domestic pig, the extent to which forage can replace the supplementary diet depends on the interaction effect between forage nutritive value and amount consumed [126]. Rivera Ferre et al. [126] calculated substitution rates of supplemental diet for forage of 4:1 and 7:1 (fresh basis) for pigs in spring and summer, respectively. Similarly, Edwards [10] obtained the same seasonal values by estimating the substitution rate with the DM intakes data presented by Mowat et al. [95] and Danielsen et al. [107]. On the other hand, swine compensatory growth can be a means to improve wild boar production by simplifying feeding strategies, improving nutrient utilisation, and improving carcass and meat quality [127].

Forage composition can also have an effect on the characteristics of carcasses and meat. Pasture grazing wild boar with restricted access to pasture typically yield leaner carcasses and have a more preferential post-morten acidification curve and therefore meat quality [128]. On the other hand, grazing pigs have a longer carcass, possibly explained by an increase in slaughter age. Pedrazzoli et al. [43] found that wild boar meat characteristics are affected by the feeding area (forest vs. farmland); the authors observed variations in lightness, colour values, tocopherol levels, index of lipid oxidation, monounsaturated fatty acids percentage, total polyunsaturated fatty acids, and intramuscular fat content. They concluded that the meat of wild boar that lived in forest had a higher oxidative stability and favourable nutritional characteristics.

## 7. Grazing Systems (Rotational vs. Continuous)

The grazing behaviour of pigs is affected by forage availability and quality, and by the time spent in a certain area [118]. When in a new area, pigs spend more time foraging than in areas that they have already spent some time in. This indicates that the two main factors that determine outdoor foraging activity are forage quality of an area and how recently the animal has entered that grazing area [97]. Pigs prefer to graze the fresher pastures compared to older areas that have recently been grazed [97]. Gustafson and Stern [86] successfully used this strategy to promote pasture consumption by offering a fresh strip of pasture every day with the additional benefit of more homogeneous manure spreading. Stern and Andresen [97] argue that the preference for certain sites indicates that pigs’ behaviour is strongly affected by the management of the area. The authors also mention that the intensive use of the recently assigned areas can have not only a nutritional purpose but also possibly also an exploratory intention, i.e., evaluating the potential of a fresh area.

Two studies tested the influence of continuous stocking (grazing continuously for 45 d in the same area), rotational grazing (7 to 10 d of grazing and 21 to 30 d of rest), and alternate grazing (alternating grazing between two paddocks, staying 15 d alternately in each) on performance [92] and behaviour [93] of finishing snout-ringed pigs, with free access to a supplementary diet. These studies did not register significant differences in animal performance (ration consumption, ADG, feed conversion, and back-fat thickness). However, they found a difference in pasture chemical composition among the different systems after 45 d [92]. Higher CP concentrations and lower ADF concentrations were found in the continuous system, compared to the two rotational systems. In turn, although the authors did not measure pasture consumption, they observed an effect on animal behaviour, since pigs in the alternate grazing system showed a higher frequency of consumption of supplementary diet than the pigs on the other two systems [93]. In addition, on the first day of observations (out of a total of three) pigs in the continuous and rotational systems showed higher grazing frequency than the pigs in the alternate system [93]. This indicates that the grazing strategy may influence the consumption patterns of domestic pigs. On the other hand, Rivero et al. [63] compared performance and behaviour of snout-ringed wild boar under continuous stocking and rotational grazing and found no difference in DM consumption, ADG or time dedicated to grazing between systems. Therefore, the grazing system selected under commercial conditions would not depend upon animal performance but should be selected based on the capabilities available at the farm and the potential environmental impact of the different grazing strategies.

## 8. Implications for Wild Boar Meat Production in Semi-Extensive Systems

The amount of forage offered is an effective tool to manipulate forage DM voluntary intake by grazing wild boars [63]. Forage inclusion levels as high as 26% of the total DM intake, which is higher than the maximum 20% recommended for pigs [122,129], can potentially be achieved without detriment to ADG and with better supplementary feed conversion than animals with no access to pasture [63], which resembles the semi-intensive production system. If similar DM consumptions were achieved between systems with and without access to pasture [63], the cost of feeding in a grazing system with a high supply of forage would be 30% cheaper, per kg of live weight gained. These calculations are based on the prices per kg DM of the supplementary diet and the forage presented by Rivero et al. [65], and the proportion of forage and supplementary diet reported by Rivero et al. [63] for the same feed conversion efficiency (3 kg DM of feed per kg LW gained [63]). This saving in feed costs, which might represent more than 60% of total costs (sum of feeding costs, non-feeding costs, and fixed costs as reported by Rivero et al. [65]), shows the advantage of including and encouraging forage consumption in the diet of growing wild boar, at least up to 26% of the total DM consumed.

Considering the information reported by Hodgkinson et al. [123], who observed that wild boar selected *P. lanceolata* over *L. perenne* from a mixed sward, and the preference study carried out by Rivero et al. [125], it can be inferred that wild boars are selective in grazing and they choose the broadleaf species and the legumes over the grasses. Therefore, it could be hypothesised that increasing the presence of the preferred species within the pasture would encourage forage consumption.

Farmed outdoor wild boars are often snout-ringed to discourage the characteristic rooting behaviour of the pigs, which can damage pasture. However, this raises animal welfare concerns and alternative strategies need to be explored. One approach may be to include below ground high-yielding crops (e.g., Jerusalem artichokes (*Helianthus tuberosus* L.) and sugar beet (*Beta vulgaris* L.)). In un-ringed growing-finishing pigs (63–86 kg LW), average daily Jerusalem artichokes intakes of 7 to 9 kg (fresh basis) have been reported corresponding to 1.3 to 1.6 kg DM [91] underpinning the potential for including root crops in diets for wild boar and domestic pigs.

The inclusion of forage in swine’ diets, in the form of direct grazing, contributes to the development of more natural productive systems, which are perceived as environmentally friendly. Semi-extensive systems can allow animals to express more natural behaviours than those in more intensive systems. Combined with the use of heritage breeds and the conditions to produce pork with favourable nutritional characteristics [130,131], this product would likely be more desirable by consumers [132,133]. Given that aspects of animal welfare and environmental sustainability are increasingly relevant, without disregarding the importance of production efficiency, moving to more sustainable production systems for swine is a priority issue.

The information available from research undertaken on grazing production systems for wild boars should be considered in the implementation of outdoor pig production systems to encourage the use of grazed pastures in domestic pigs’ habitat and diet, as well as increasing the nutrients consumed from pasture and improving animal welfare. This would be essential to increase the contribution of pasture to outdoor pig production systems, not only as a “natural” environment to raise pigs but also as a significant source of good quality and low-cost nutrients. Therefore, further studies on the dietary requirements of wild boar and feed conversion ratio are needed because feed is the most relevant production cost for farmers. The long-term sustainability of wild boar production is dependent on the economic, social, and environmental viability of the systems. Pasture-based production systems may be one way by which this can be achieved, but only if implemented in the right manner. Achieving this requires continued research on wild-boar production systems and how their performance can be improved in relation to all of these factors.

## Figures and Tables

**Table 1:** Foraging behaviour and daily dry matter (DM) intake of wild boar and domesticated pigs with access to pasture (review of 22 studies from 2001 to 2018).

Pig Type/Pig Breed	Liveweight Range (kg)	Initial Age	Concentrate Level (kg DM/d)	Pasture Crop	Rooting (% Time)	Grazing (% Time)	Daily Pasture DMI (g/d)	Daily Total DMI (g/d)	Daily Gain (g/d)	F:G	Ref.
PR	40–90		0.088 × BW^0.75^ 0.061 × BW^0.75^	Legumes/forbs			880 1550	2920 2920	646 527	3.14 2.64	[76]
PR	14.5 48.6		R-85%	Clover/forbs			97 208			2.66 3.03	[84]
DP	30–60		1.26	Grass/clover			372	1710	544	3.16	[85]
LW × LL	30–110	76 d	1.4–2.8 1.6–3.2	Grass/clover			307 219	2121 2162	879 912	3.2 3.3	[86]
(LL × LW) × DD	58–90		≈2.2 kg (20.5 vs. 10.7% CP)	Grass/lucerne	15.5–44.4	3.6–10.6	330–470 lucerne		589–900	2.5–3.8	[87]
LW × LL	27	56 d	R-80%	Grass (kikuyu)			102 (OM)	1094 (OM)			[88]
(LL × YY) × DD (LL × YY) TT	33.7	88 d	50%, 80%, 115% NA	Grass clover w. herbs/root chicory	18–40%	6–9%		1750–2000 (conc.)	648–863	2.78–3.25	[89,90]
(LL × YY) × DD	61–110 64–86	126 d	Ad libitum vs. R	Jerusalem artichokes	3.9% 4.5%	1.1% 7.9%		4260 (conc.) 1039 (conc.)	1224 560	3.50 1.66	[91]
(LW × LL) × DD	27–90		Ad libitum	Clover	2.23%	5.65%		2370–2440 (conc.)	800–830	2.92–2.98	[92,93]
CS × LW + hybrid	40–150		Indoor Free-range Organic						920 860 760	2.70 3.11 2.90	[94]
CB × DD	56		Ad libitum	Grass/clover			100 (OM)	2290 (OM)			[95]
Iberian	112		None	Dehesa: Oaks and shrubs + grassland		61.3–71.2%	380–490 (grass) 3090–3630 (AK)	3470–4120	760	5.23	[20,51]
Iberian	110–158	12 m	None	Dehesa: Oaks and shrubs + grassland		61.5%	500 (grass) 2900 (AK)	3400	790	4.00 (AK) + 0.7 (grass)	[96]
LL × LW	50–81	14 w	NA 80% NA	Grass/clover	5.8% 8.5%	30.0% 33.6%		811 (conc.) 868 (conc.)	812 686	3.36 3.19	[97]
WB (LW × LL) × WB	15–58 54–242	81 d 81 d	R1AG	Grass/clover	6.4% 0.6%	37% 17%	354 446	1536 4090	179.8 758	3.7 2.7	[6]
WB (LL × LW) × WB	10 31	3 m	R1AG	Grass/clover			87 70	667 1635	198 608	2.7 2.8	[98]
WB	18.8		R1AG	Grass Forb			210–418 226–550		227		[53]
WB	14.4	120 d	R1AG	No pasture Grass/clover			- 158	671 (conc.) 1007	224 303	3.33 2.33	[63]
WB	18.3		R1AG	Grass/clover		42%	242		251	2.47	[62]

DMI: DM intake, F:G: Supplemental feed (DM) to gain ratio (unless other feed stated), PR: Pampa Rocha, DP: Domestic pig, LW: Large White, LL: Landrace, DD: Duroc, YY: Yorkshire, TT: Tamworth, CS: Cinta Senese, CB: Camborough, WB: wild boar, BW: body weight, R: restricted, CP: crude protein, NA: normal allowance, R1AG: Restricted to 1 h after grazing, OM: organic matter, AK: acorn kernel, conc.: concentrate.
